# Inverted Meckel’s Diverticulum: A Rare Cause of Intussusception

**DOI:** 10.7759/cureus.94934

**Published:** 2025-10-19

**Authors:** Faraz Sharif, Muhammad Raheel, Constantine Ezeme, Daniel Thomas, Chris Macklin

**Affiliations:** 1 General and Colorectal Surgery, Mid Yorkshire Teaching NHS Trust, Wakefield, GBR

**Keywords:** intussusception, inverted meckel’s diverticulum, laparoscopic wedge resection, lead point, meckel’s diverticulum, small bowel obstruction

## Abstract

Meckel’s diverticulum (MD) is the most common congenital anomaly of the gastrointestinal tract. Rarely, MD has been noted to invaginate, which causes a pathological lead point for intussusception and causes small bowel obstruction. Diagnosing inverted Meckel’s diverticulum (IMD) can be difficult due to its rarity, vague presentation, and limitations of conventional imaging modalities in picking it up.

A 56-year-old woman was referred to the Gastroenterology clinic after a recent hospital admission for infectious gastroenteritis. She reported being generally well with few symptoms. She had a tendency to constipate, opening her bowels only once in every 5-6 days. Computed tomography of the abdomen and pelvis with contrast showed a 4 cm mid small bowel polyp with an 8 cm stalk, thought to be a lipoma. Oral route double balloon enteroscopy failed as the polyp was not accessible due to its location. She underwent elective laparoscopy, which showed an intussuscepting polyp at the proximal ileum, and wedge resection of the proximal ileum was performed with an intestinal stapler with side-to-side anastomosis. Histology showed MD, which inverted into the small bowel lumen.

This case highlights the need to consider IMD in the differential diagnosis of unexplained gastrointestinal symptoms, particularly in middle-aged and older adults. Increased awareness of this rare entity can facilitate early recognition and timely intervention, ultimately improving patient outcomes.

## Introduction

Meckel’s diverticulum (MD) is the most common congenital anomaly of the gastrointestinal tract, with a prevalence of around 1.2% [1]. It is a true diverticulum that contains all three layers of the gut wall and often contains ectopic tissue, such as gastric, duodenal, or pancreatic tissue [1]. Although most cases tend to remain asymptomatic, it can cause complications including inflammation, obstruction, haemorrhage and perforation [2]. Rarely, MD has been noted to invaginate, which causes a pathological lead point for intussusception and causes small bowel obstruction. Very few cases have been reported in the literature, with only one case previously being reported in the United Kingdom [1].

Diagnosing inverted Meckel’s diverticulum (IMD) is challenging due to its rarity, nonspecific presentation, and the limited sensitivity of conventional imaging. Furthermore, there is no standardised management approach, with treatment ranging from endoscopic to surgical interventions.

We report a case of a 56-year-old woman with IMD, highlighting the diagnostic challenges, clinical considerations, and therapeutic options associated with this uncommon entity.

## Case presentation

**Figure 1:**
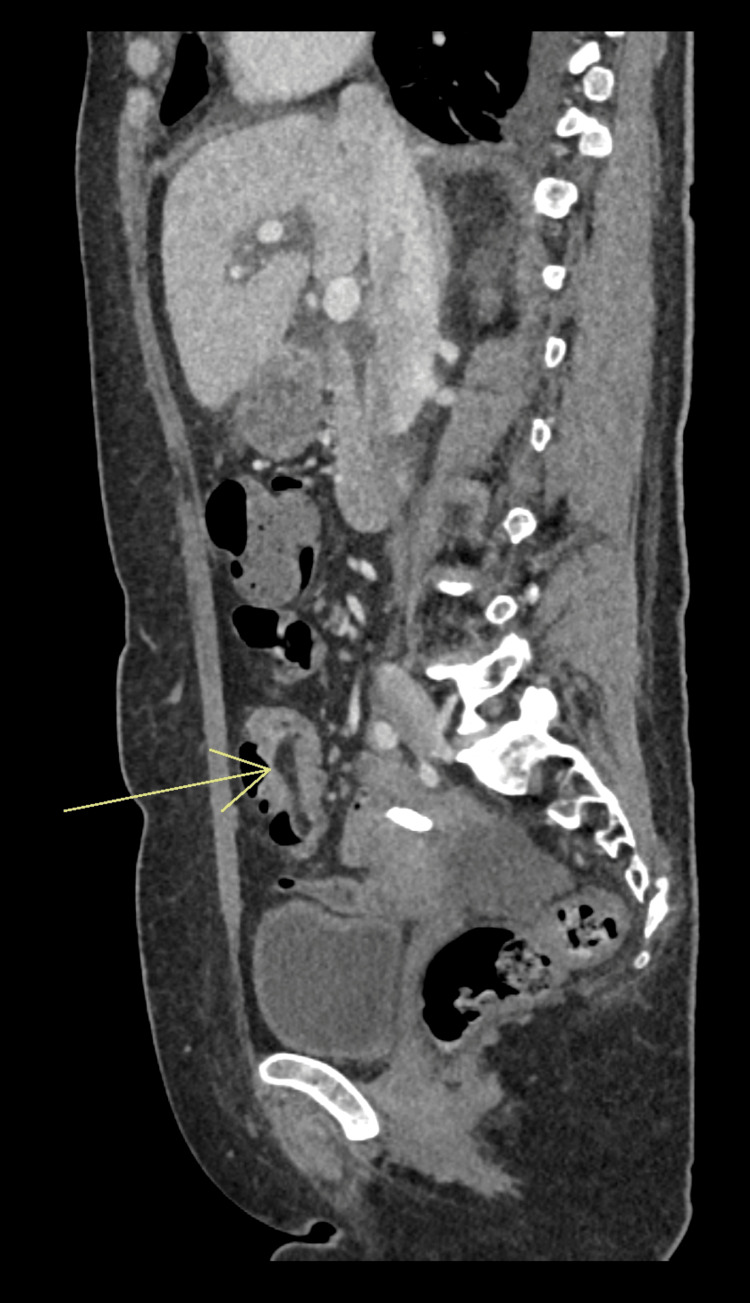
Sagittal view of the polyp shown on CT scan abdomen with contrast

**Figure 2:**
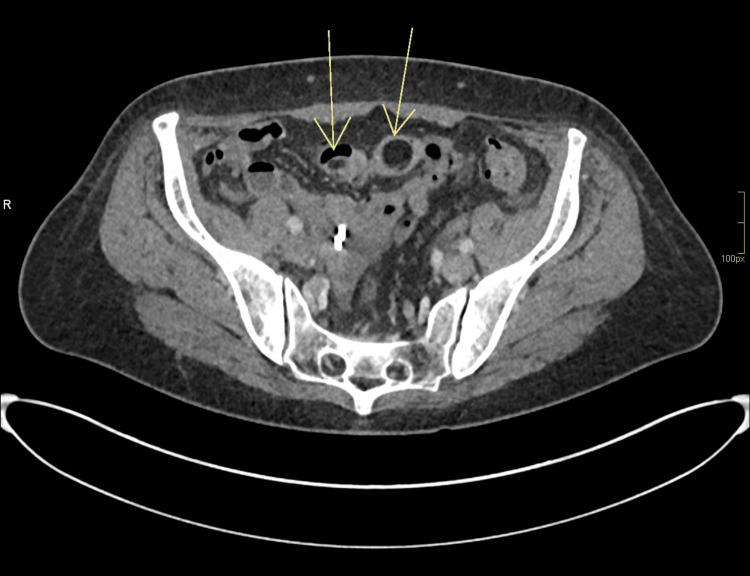
Axial view of the polyp shown on CT scan abdomen with contrast

**Figure 3:**
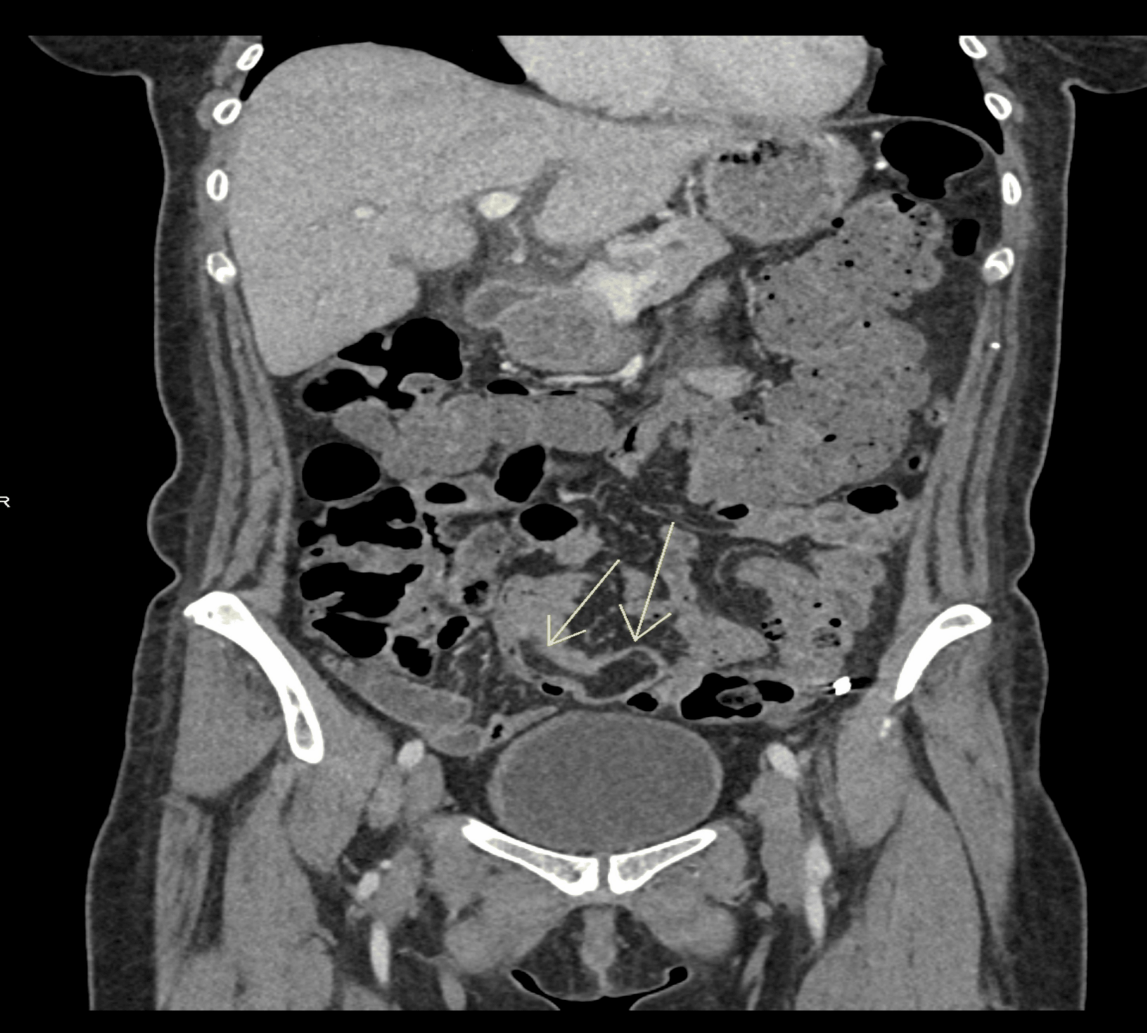
Coronal view of the polyp shown on CT scan abdomen with contrast

A 56-year-old woman was referred to the Gastroenterology clinic following a recent hospital admission for infectious gastroenteritis. During that admission, a contrast-enhanced CT of the abdomen and pelvis incidentally revealed a 4 cm polypoid lesion with an 8 cm stalk within the pelvis (Figures 1-3), initially thought to represent a lipoma. The scan also noted that the lesion appeared to act as a lead point for a short segment of small bowel intussusception, with no evidence of obstruction. Given the chronic and stable appearance of the findings, she was discharged for outpatient follow-up.

Interestingly, this abnormality had been documented on an outpatient CT scan performed four years earlier, but the finding was not acknowledged at the time and no further action was taken.

She was seen in the clinic six months after hospital admission. She had a background of JAK-2 positive essential thrombocythemia, epilepsy and pancreatitis with no relevant family history. She had no previous abdominal surgery. She reported being generally well, though she experienced chronic constipation, opening her bowels every 5-6 days, along with intermittent bloating and mild upper abdominal discomfort. These symptoms were persistent but non-debilitating and had minimal impact on her daily activities, with no nausea, vomiting, weight loss, or appetite change.

An outpatient oral route double balloon enteroscopy (DBE) was performed. The procedure was difficult with the scope progressing to the proximal to mid-ileum but unable to reach the polyp. In places, the lumen looked dilated, suggesting chronic stasis but the lumen was empty. She was referred to the General Surgery team for surgical intervention.

**Figure 4:**
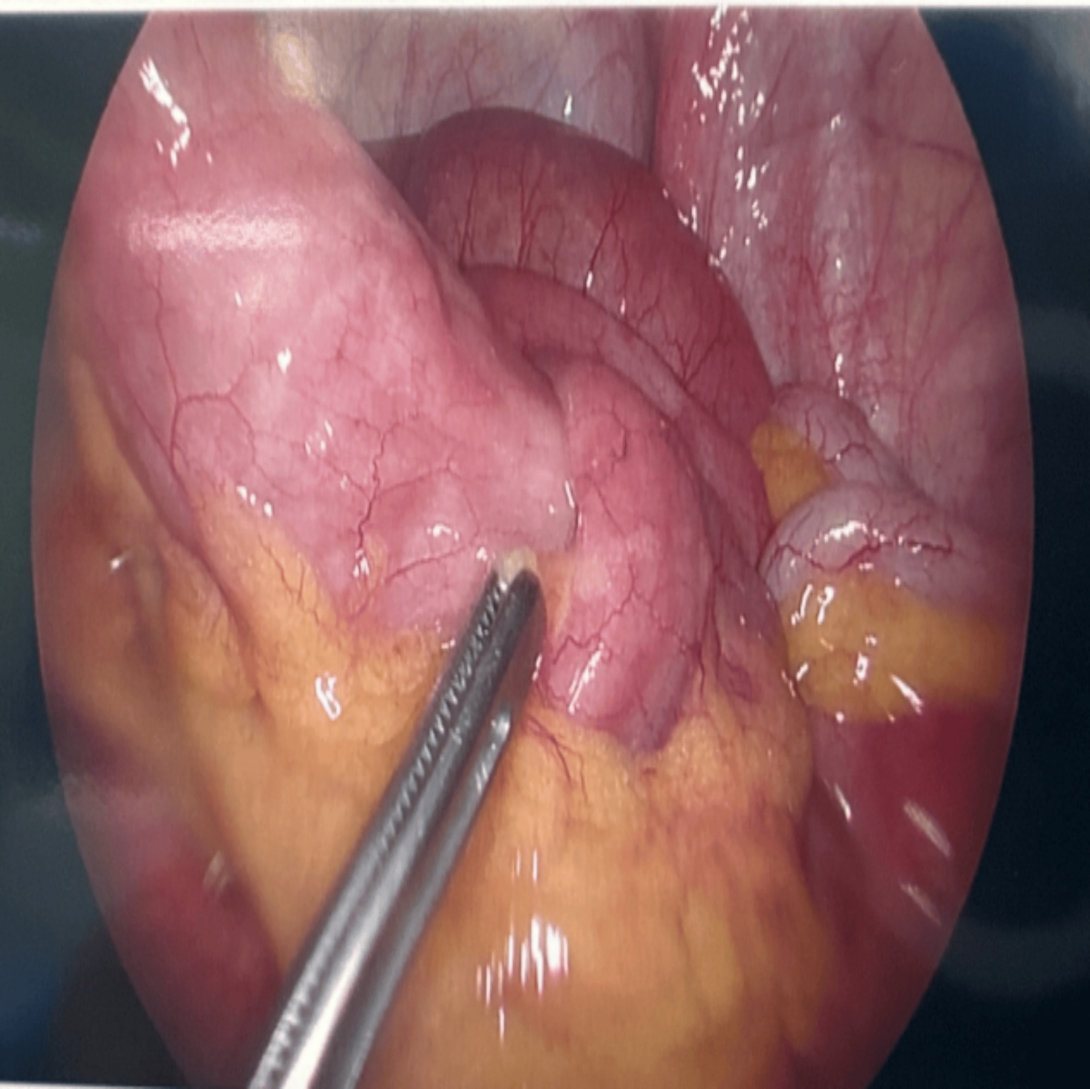
Intra-operative laparoscopic view of intussucepting polyp

**Figure 5:**
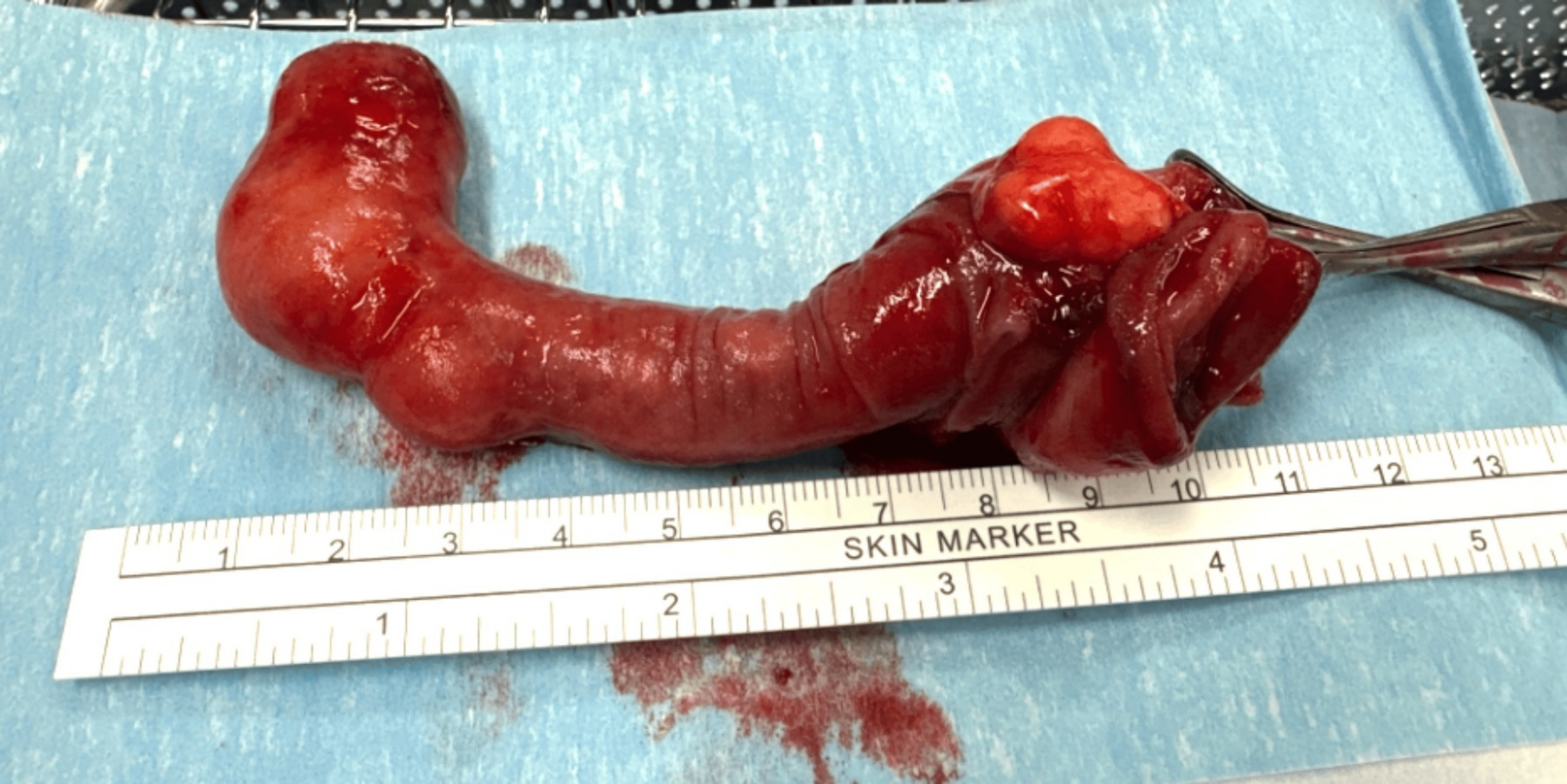
Polyp seen after excision with a skin marker ruler

Four months after her enteroscopy, she had elective laparoscopy which showed an intussuscepting polyp at the proximal ileum (Figures 4, 5). Wedge excision of the proximal ileum was performed with an intestinal stapler with side-to-side anastomosis. Her admission was uneventful with a post-operative stay of five days. At two-month follow-up, she was symptom-free and had experienced no post-operative complications.

**Figure 6:**
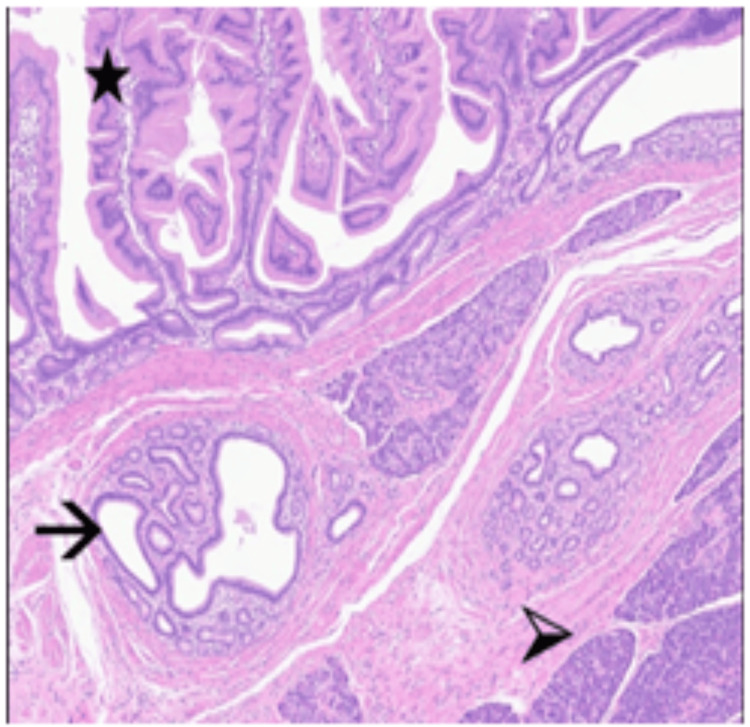
Heterotopic gastric foveolar epithelium (*) and heterotopic pancreatic tissue comprising ducts (->) and lobules of pancreatic acini (>). The appearances are in keeping with a Meckel’s diverticulum

Histology showed indrawing of the mesenteric fat and vessels. This was predominantly surfaced by small bowel mucosa, but there was a submucosal mass of heterotopic pancreatic tissue with organised lobules of acini, small groups of islet cells and bland ducts (Figure 6). There was an overlying gastric foveolar epithelium, which connected with the luminal small bowel epithelium. Whilst there was a component of mature fat, the appearances raised the possibility that this may have been an underlying Meckel's diverticulum, which had inverted into the small bowel lumen. There was no evidence of dysplasia or malignancy, and the margins were clear with normal muscularis and mucosa.

## Discussion

MD occurs due to the incomplete obliteration of the vitellointestinal duct during fetal development. It is commonly known by the rule of twos: occurring in 2% of the population, located two feet from the ileocaecal valve, and typically measuring 2 inches in length [2]. The clinical presentation of MD is variable, ranging from an absence of symptoms to occasional abdominal discomfort to complications including haemorrhage, MD, chronic ulceration, intestinal obstruction, perforation, and intussusception [2].

Inversion of MD is an uncommon complication. The precise mechanism remains uncertain, with proposed theories including abnormal peristaltic activity, adhesions, or fibrous bands anchoring the diverticulum [3]. Inversion can create a pathological lead point for intussusception. Acute cases present with symptoms of bowel obstruction such as abdominal pain, bloating, vomiting, and altered bowel habits, occasionally accompanied by gastrointestinal bleeding (haematochezia or melaena) [4]. In contrast, chronic or intermittent presentations may involve colicky pain, constipation, anaemia, or weight loss, often leading to delayed diagnosis [4].

In our case, the presentation was chronic and insidious, marked by mild constipation and intermittent abdominal discomfort, consistent with other reported cases of inverted Meckel’s diverticulum presenting without acute obstruction. The initial CT findings were misattributed to a benign lipoma and overlooked for several years, a delay similar to reports in the literature where incidental findings on imaging were not recognised until subsequent review. This case therefore underscores the diagnostic challenge and the potential for missed or delayed recognition when findings occur in asymptomatic or minimally symptomatic patients.

Imaging remains the first step in evaluation. CT is most commonly used, particularly in non-acute presentations [5]. It is useful to diagnose complications such as obstruction, intussusception or diverticulitis but still not sensitive in diagnosing MD. CT and MR enterography are newer innovations that allow improved visualisation of the small bowel wall, improving the sensitivity of diagnosis [5]. Abdominal X-ray can show features of obstruction and ultrasonography can detect intussusception but they are otherwise of limited clinical value. Scintigraphy with 99mTc-Na-pertechnetate is highly specific for identifying ectopic gastric mucosa, which is commonly found in MD [6]. In our patient, the diagnosis was not established radiologically despite multiple imaging studies, reflecting the limitations described in the literature.

Definitive diagnosis often requires intra-operative evaluation for biopsy. Gastroscopy is typically unremarkable. Capsule endoscopy offers a minimally invasive method of evaluating the small bowel and may incidentally identify an IMD [7]. Deep enteroscopy techniques such as single-balloon enteroscopy and DBE may allow for direct visualisation, with DBE being preferable due to its superior reach into the small bowel [8]. However, even with DBE, the lesion may still not be accessible, as in this index case. It also offers the potential of therapeutic intervention with several case reports showing endoscopic snaring as a treatment in case of a polyp. Limiting factors in endoscopic snaring include poor access, size constraints, and the risk of incomplete resection [9]. In our case, endoscopic management was not possible, prompting surgical referral.

Surgical resection remains the mainstay of IMD management, performed via either laparoscopic or open approaches. Laparoscopy is preferred due to its minimally invasive nature, with associated shorter hospital stays and reduced post-operative morbidity. The surgical option for IMD is resection of the involved bowel segment and anastomosis. Following surgical excision, long-term outcomes are excellent, with minimal risk of post-operative complications [10].

Post-operative histopathological analysis is important to confirm the diagnosis and exclude malignancy, although malignant transformation of MD is rare [11]. In approximately 20% of cases, its mucosa may contain ectopic gastric, colonic, or pancreatic tissue [2]. The presence of these tissues increases the risk of mucosal irritation, chronic inflammation, or ulceration and the subsequent risk of haemorrhage and intussusception.

In summary, this case reinforces several key observations from the literature: IMD often presents with non-specific or chronic gastrointestinal symptoms, may be missed on imaging, and typically requires surgical resection for definitive management. Recognition of incidental findings, timely multidisciplinary review, and awareness of this rare entity can help prevent diagnostic delays and improve patient outcomes.

## Conclusions

This case underscores the importance of considering IMD in the differential diagnosis of unexplained or chronic gastrointestinal symptoms, particularly in middle-aged and older adults. It highlights how incidental findings may be overlooked when imaging is performed for unrelated reasons and reviewed outside of specialist settings.

Clinicians should remain vigilant in reviewing prior imaging and reassessing historical findings that may have ongoing relevance. Early multidisciplinary discussion and timely surgical consideration are key when IMD or other pathological lead points are suspected, as delayed recognition can prolong symptoms and increase the risk of complications. Greater clinical awareness of this rare entity and its variable presentation can therefore facilitate earlier diagnosis, appropriate intervention, and improved patient outcomes.
